# Celastrol Attenuates FGF23 Expression in Osteoblasts by Inhibiting STAT3 Activation

**DOI:** 10.2174/0118715303420913251205154844

**Published:** 2026-07-01

**Authors:** Jiawei Cao, Yuewen Tang, Feng Wan, Tingjiao Xu, Ruchun Yang

**Affiliations:** 1 Department of Nephrology, Hangzhou TCM Hospital Affiliated to Zhejiang Chinese Medical University, Hangzhou, China;; 2 Department of Nephrology, Hangzhou Dingqiao Hospital, Hangzhou, China;; 3 Zhejiang Key Laboratory of Blood-Stasis-Toxin Syndrome, Zhejiang Chinese Medical University, Hangzhou, China;; 4 College of Life Sciences, Zhejiang Chinese Medical University, Hangzhou, China

**Keywords:** Extracellular phosphate, FGF23, STAT3, celastrol, CKD, osteoblasts

## Abstract

**Introduction:**

In this study, we aim to investigate the mechanism by which extracellular phosphate (Pi) regulates the transcription of fibroblast growth factor 23 (FGF23) in osteoblasts and the effects of celastrol on FGF23 expression in UMR-106 cells and in chronic kidney disease (CKD) rat models.

**Methods:**

We used Pi to induce FGF23 expression in a rat osteoblastic cell line as a model and then treated the cells with celastrol. The cytotoxicity of Pi and celastrol was assessed using the CCK-8 assay. FGF23 expression was measured using real-time quantitative PCR (RT-qPCR), and its promoter activity was evaluated using luciferase reporter assays. To detect active signal transducer and activator of transcription 3 (STAT3), immunofluorescence and Western blotting were performed. The 5/6 nephrectomized rat model was established to investigate the effects of celastrol on FGF23 expression *in vivo*. An enzyme-linked immunosorbent assay was carried out to examine serum FGF23 levels in rats. RT-qPCR detected the mRNA levels of FGF23 and STAT3 in the femurs of CKD rats.

**Results:**

We found that high Pi activated STAT3 and promoted its nuclear translocation, leading to elevated FGF23 expression, which was suppressed by celastrol. Additionally, in rats subjected to 5/6 nephrectomy with high serum Pi levels, serum FGF23 levels and skeletal STAT3 expression were significantly decreased after celastrol treatment.

**Discussion:**

Therefore, celastrol could have therapeutic effects on CKD by decreasing serum FGF23.

**Conclusion:**

High Pi activates STAT3 and induces FGF23 expression. Furthermore, celastrol can attenuate increased FGF23 expression induced by high Pi, potentially *via* inhibition of STAT3 activation.

## 1 INTRODUCTION

The prevalence of chronic kidney disease (CKD) among adults in China stands at 8.2%, ranking first worldwide, highlighting its public health burden and the need for early intervention [1]. Elevated serum fibroblast growth factor 23 (FGF23) is an independent risk factor for mortality in CKD [2, 3]. Furthermore, FGF23 levels are closely associated with CKD progression and its complications [4-6]. FGF23, primarily secreted by osteoblasts and osteocytes, is a key regulator of serum phosphate (Pi) levels [7]. In end-stage renal disease, when renal function is severely reduced, abnormally elevated FGF23 expression occurs concurrently with hyperphosphatemia [8]. Moreover, elevated dietary Pi levels are associated with higher FGF23 levels in humans and animals, suggesting that Pi may upregulate FGF23 expression [9, 10]. *In vitro*, extracellular Pi regulates gene expression in various skeletal and non-skeletal cells [11, 12]. However, the molecular basis of FGF23 expression regulation in response to alterations in serum Pi levels remains unclear. As a transcription factor, signal transducer and activator of transcription 3 (STAT3) is essential for various biological processes, including sensing and responding to extracellular signals [13, 14]. Based on our studies, we hypothesized that extracellular Pi activated STAT3 and promoted FGF23 expression in osteoblasts.

Traditional Chinese medicine shows positive clinical outcomes in the treatment of kidney diseases [15, 16]. Celastrol, an active component of the Asian perennial vine *Tripterygium wilfordii*, is known for its anti-inflammatory, antioxidant, and anticancer properties [17-19]. Given the central roles of inflammation and oxidative stress in the progression of CKD [20], celastrol has emerged as a promising therapeutic agent in experimental models [21, 22]. Furthermore, it can inhibit STAT3 activation, as previously reported [23]. Therefore, we propose that celastrol may attenuate FGF23 expression by inhibiting STAT3 activation.

This study aimed to uncover the mechanism by which celastrol attenuates Pi-induced FGF23 expression through the inhibition of STAT3 activation. The effects of Pi and celastrol on STAT3 phosphorylation levels and FGF23 transcriptional activity were examined in a rat osteosarcoma cell line (UMR-106). Additionally, a 5/6 nephrectomized rat model was established to evaluate the impact of celastrol on STAT3 and FGF23 expression. This study preliminarily elucidated the pharmacological mechanism of celastrol in reducing FGF23 levels.

## 2 MATERIALS AND METHODS

### 2.1 Reagents and Drugs

Pi buffer (0.1 M) was adjusted to pH 7.4, containing Na_2_HPO_4_ (S112619, Aladdin, China) and NaH_2_PO_4_ (S102313, Aladdin, China) in the molar ratio 4:1, and was used for Pi stimulation. The other materials included the following: celastrol (Chengdu Refensi Biotechnology Co., Ltd, China), NSC 74859 (SD4794, Beyotime, China), Na_2_SO_4_ (S110746, Aladdin, China), TRIzol reagent (9109, Takara, Japan), Primescript™ reagent kit (RR036A, TaKaRa, Japan), TB Green Premix Taq (RR420A, Takara, Japan), rat FGF23 enzyme-linked immunosorbent assay (ELISA) Kit (CSB-E12170r, CUSABIO, China), fetal bovine serum (A5669701, Gibco, USA), PEI MAX (24765, Polysciences, USA), Cell Counting Kit-8 (GK10001, GLPBIO, USA), Dual-Luciferase^®^ Reporter Assay System (E1910, Promega, USA), bicinchoninic acid protein assay kit (P0010, Beyotime, China), anti-STAT3 antibody (9139, Cell Signaling Technology, USA; AF1492, Beyotime, China), anti-phospho-STAT3 antibody (AF1276, Beyotime, China), anti-Myc tag antibody (AF2864, Beyotime, China), anti-GAPDH antibody (60004-1-Ig, Proteintech, China), IRDye 800CW, and 680RD secondary antibody for Western blotting (926-32210, 926-68071, LI-COR, USA), Alexa 546 conjugated secondary antibody for Immunofluorescence (A11030, Invitrogen, USA), DAPI (G1012, Servicebio, China), and BeyoGold™ His-tag Purification Resin (P2233,Beyotime, China), FastDigest BamHI (FD0054, Thermo Scientific, USA), and ClonExpress II One Step Cloning Kit (C112-01, Vazyme, China).

### 2.2 Animals

The animal research was carried out in compliance with the ARRIVE guidelines and received approval from the Institutional Animal Care and Use Committee at Hangzhou Medical College (approval no. ZJCLA-IACUC-20070015) [24]. Eighteen male Sprague–Dawley rats (6 weeks old, weight 190-210 g) were obtained from Hangzhou Medical College. The rats were kept at Hangzhou Medical College Laboratory Animal Center, where they were subjected to a diurnal cycle consisting of 12 hours of light and 12 hours of darkness. Throughout the experiment, the rats were housed in a controlled environment (23°C, 60% humidity) and allowed unrestricted access to balanced feed and water.

### 2.3 Experimental Design

Following two weeks of environmental acclimation, the rats were divided into the control (n = 6) and model (n = 12) groups at random. The sample size was approved by the Institutional Animal Care and Use Committee at Hangzhou Medical College. The control rats underwent a sham surgery. CKD was induced in the model group by performing a 5/6 nephrectomy. Rats underwent 5/6 nephrectomy under anesthesia with 1.0% pentobarbital sodium (45 mg/kg body weight). In the first surgical step, the right kidney of the rats was surgically removed. One week after uninephrectomy, a standardized subtotal nephrectomy was conducted, excising approximately 2/3 of the left renal cortex. A single experimenter performed all surgeries to reduce inter-operator variability. Four weeks after nephrectomy, the rats were divided into two groups as follows: (1) six rats in the model group, and (2) six rats were given celastrol (3 mg/kg body weight) *via* intragastric administration once daily in the celastrol group. The concentration of celastrol used *in vivo* was optimized based on prior studies to avoid adverse effects [25, 26].

### 2.4 Sample Collection

After 8 weeks, the rats were anesthetized *via* intraperitoneal injection of sodium pentobarbital (35 mg/kg). Subsequently, serum and femurs were collected from all groups and stored at -80°C.

### 2.5 RNA Extraction and RT-qPCR

Bone samples were pulverized using a bead-beater homogenizer (Jingxin, China). Isolation of total RNA from bone samples and UMR-106 cells was performed using TRIzol reagent, followed by reverse transcription into cDNA for RT-qPCR analysis. Total RNA (1 μg) was reverse-transcribed into cDNA using the PrimeScript™ Reagent Kit with gDNA Eraser. RT-PCR assays were performed on a QuantStudio 5 Real-Time PCR System (Applied Biosystems, USA) using TB Green Premix Taq. The 20 μl RT-qPCR reaction mixture consisted of 100 ng of cDNA, 10 μl TB Green Premix Taq, 0.5 μM of each forward and reverse primer, and sterile water to volume. Amplification was performed with an initial hold at 95°C for 35 s, followed by 40 cycles of 95°C for 5 s and 60°C for 30 s, and a final melt curve stage to verify the specificity of the PCR products. The relative mRNA expression of FGF23 and STAT3, normalized to *Gapdh*, was calculated by the 2^–ΔΔCT^ method. The primers utilized in this investigation are detailed in Table **1**. The experiment was performed with three independent biological replicates.

### 2.6 ELISA

Rat blood samples were centrifuged at 3000 rpm for 10 minutes at 4°C to separate the serum. The harvested serum was aliquoted and stored at -80°C for further analysis. Rat ELISA kits were used to measure serum FGF23 levels, following the manufacturer’s protocol. Briefly, samples and standards were loaded onto the pre-coated plate. After incubation and washes, a biotin-labeled antibody, an HRP-Streptavidin conjugate, and a TMB substrate were added sequentially. Absorbance was measured at 450 nm, and FGF23 concentrations were calculated from the standard curve based on sample OD values.

### 2.7 Cell Culture and Treatment

The UMR-106 cell line was obtained from the Cell Bank of the Chinese Academy of Sciences and maintained in DMEM containing 10% fetal bovine serum (FBS) at 37°C in a 5% CO_2_ atmosphere. The doubling time of the UMR-106 cells was approximately 24 hours. All experiments were performed using cells between passage numbers 5 and 15 to ensure phenotypic stability. Cells were seeded into 6-well plates at a density of 2 × 10^5^ cells per well and starved in a medium with 2% FBS for 12 hours before being treated with multiple reagents for 24 hours. UMR-106 cells were divided into four groups: control group (Ctrl), 3 mM Pi (Pi), 3 mM Pi + 300 nM celastrol (Pi + Cel), and 3 mM Pi + 50 μM NSC 74859 (Pi + NSC). Cells were transfected with pLV3-CMV-FGF23-Myc-His plasmids when they reached 50% confluence. For FGF23 mRNA detection, all cells were treated with 1 nM calcitriol to induce FGF23 expression, which was undetectable under basal conditions. For the CCK-8 assay, cells were treated with varying concentrations of Pi or celastrol for 24 hours following starvation. Then, the medium was replaced with a CCK-8 working solution. After incubation for 1–4 hours at 37°C, the absorbance at 450 nm was then determined. The osmolarity of the cell culture medium was measured using the freezing-point depression method on an Osmomat 030 (Gonotec, Germany). All measurements were performed in triplicate.

### 2.8 Plasmid Constructs

The expression plasmid for rat FGF23 (pLV3-CMV-FGF23-Myc-His) was generated by standard molecular biology techniques. The template cDNA was extracted from UMR-106 cells. The vector was linearized by BamHI digestion, generating homologous arms at the insertion site. The FGF23 gene was PCR-amplified from cDNA with primers containing extensions homologous to the vector ends (F: 5′- AATTCGAATTTAAATGGATCCGCCACCATGCTGGG GGCCTGCCTC -3′; R: 5′- ATGGTGATGGTGATGGGATCCGACGAACCTGGGAAAGGGG -3′).

### 2.9 Luciferase Reporter Assays

The FGF23 promoter fragment was selected based on previous studies [27]. UMR-106 cells were co-transfected with GV715-FGF23-p-Luc, containing the FGF23 promoter (NM_130754, ~2000 bp) and pRL-TK-luc in a 50:1 ratio. The luciferase reporter plasmid was constructed by GeneChem Co., Ltd. (Shanghai, China). UMR-106 cells on 24-well plates were transfected with the reporter vector (0.5 μg) and pRL-TK-luci (0.01 μg) by PEI. At 12 hours post-transfection, the cells were treated with Pi, celastrol, or NSC 74859 for 24 hours. Luciferase activities were then assessed.

### 2.10 Immunofluorescence

Cells were incubated in 4% paraformaldehyde for 30 minutes at room temperature for fixation, and goat serum (5%) was used for blocking. Subsequently, cells were treated with an anti-STAT3 antibody and incubated at 4°C overnight. Alexa 546 conjugated secondary antibody was incubated for 1 hour. After nuclear staining with DAPI, imaging was conducted using a high-content analysis system (Operetta CLS, PerkinElmer, USA) and analyzed using ImageJ software.

### 2.11 Immunoprecipitation

UMR-106 cells were seeded in 60-mm dishes at a density of 1 × 10^6^ cells per dish. After a 48-hour transfection period, the cell culture supernatant was collected and centrifuged at 2000 × g for 10 minutes at 4°C to remove cellular debris, while the cells were harvested and lysed for subsequent analysis. For each reaction, 10 μL of His-tag purification resin was incubated with 4 ml of supernatant for 4 hours at 4°C with gentle rotation. The resins were collected and washed three times with PBS supplemented with protease and phosphatase inhibitors. The immunoprecipitates were eluted with RIPA buffer (50 mM Tris at pH 7.4, 150 mM NaCl, 1% (v/v) Triton X-100, 1% (v/v) sodium deoxycholate, 0.1% (v/v) SDS) for further analysis.

### 2.12 Western Blotting

Lysed cells were processed with RIPA buffer. The buffer was supplemented with protease and phosphatase inhibitors. A bicinchoninic acid protein assay kit was used to determine the total protein concentration. Denatured proteins were heated at 100°C for 10 minutes, then subjected to electrophoresis and transferred to a cellulose filter membrane. Membranes were incubated with different antibodies and visualized through an Odyssey infrared imaging system (LI- COR Biosciences, USA). The grayscale analysis was conducted using ImageJ.

### 2.13 Statistical Analysis

The results are presented as the mean ± standard deviation. Data analysis was conducted using GraphPad Prism software (version 8.0.1). Significant differences among groups were assessed using one-way ANOVA, with Tukey’s post hoc test employed for multiple comparisons. *P* < 0.05 was considered statistically significant.

## 3 RESULTS

### 3.1 High Extracellular Pi Upregulates FGF23 mRNA Levels and Increases Active STAT3 Expression

In bodily fluids, Pi mainly exists in the form of H_2_PO_4_^−^/HPO_4_^2−^ at a 1:4 molar ratio; therefore, the same ratio was used to prepare the Pi solution. We added different concentrations of Pi to the medium of rat osteoblast UMR-106 cells to simulate the hyperphosphatemia observed in patients with CKD. Using the CCK-8 assay, we confirmed that there was no significant cytotoxicity at Pi concentrations below 12.5 mM (Fig. **1A**). Furthermore, serum Pi levels can affect FGF23 levels in patients with CKD [28]. Therefore, we detected FGF23 expression in UMR-106 cells using RT-qPCR and found that it was significantly induced by 3 mM Pi (Fig. **1B**). To overcome the low basal FGF23 mRNA expression, cells were treated with 1 nM calcitriol to induce transcriptional activation as previously described [29]. As commonly reported in the literature, STAT3 enhanced transcription of the FGF23 promoter [27]. Thus, we detected active STAT3 in UMR-106 cells following Pi stimulation. As demonstrated by immunoblotting, elevated extracellular Pi triggered STAT3 phosphorylation in a dose-dependent manner (Fig. **1C**). Moreover, with the extension of the treatment time, the level of STAT3 phosphorylation increased (Fig. **1D**). To exclude the osmotic influence of Pi, we measured the osmolarity of the cell culture medium. The control medium consisted of DMEM supplemented with 10% FBS. The osmolalities of the control medium, the medium supplemented with 3 mM Pi, and the medium supplemented with 3 mM Na_2_SO_4_ were 344.3 ± 0.6, 346.0 ± 1.7, and 345.0 ± 2.0 mOsm/kg, respectively (Supplementary Table **S1**). Subsequently, we confirmed that the addition of Na_2_SO_4_, despite altering osmolarity to a similar extent as Pi, induced neither FGF23 expression nor STAT3 phosphorylation (Figs. **1E** and **F**). Based on these findings, 3 mM Pi was used to induce FGF23 expression in subsequent experiments. Collectively, these findings indicated that FGF23 gene expression and STAT3 phosphorylation were elevated in response to high extracellular Pi.

### 3.2 Celastrol Inhibits STAT3 Phosphorylation in Pi-treated Osteoblasts

Celastrol is a bioactive terpenoid that contributes to the pharmacological activity of the Chinese herb *T. wilfordii* (Fig. **2A**). Moreover, it appears to inhibit the activity of STAT3 [30]. In our study, we confirmed that celastrol concentrations below 625 nM were not cytotoxic to osteoblasts (Fig. **2B**). We treated Pi-induced osteoblasts with celastrol or the STAT3 inhibitor NSC 74859 and observed that both could decrease the STAT3 phosphorylation levels (Fig. **2C**). As phosphorylated STAT3 entered the nucleus to promote gene transcription, we also detected STAT3 nuclear translocation after celastrol or NSC 74859 treatment *via* immunofluorescence. Nuclear STAT3 was reduced in the cells treated with celastrol or NSC 74859 (Fig. **2D**). This finding suggested that celastrol inhibited the activation of STAT3 induced by Pi in osteoblasts.

### 3.3 Celastrol Downregulates the Expression of FGF23 *via* STAT3 in Osteoblasts

The FGF23 expression induced by extracellular Pi was inhibited by celastrol, as well as NSC 74859 (Fig. **3A**). There are multiple STAT3-binding sites in the FGF23 promoter. Therefore, we transfected UMR-106 cells with a dual-luciferase reporter vector containing the 2000 bp promoter region upstream of the rat FGF23 gene. As shown in Fig. (**3**), celastrol inhibited transcriptional activity of the FGF23 gene (Fig. **3B**). This finding indicated that celastrol might reduce FGF23 expression by inhibiting STAT3 activation. Since FGF23 is an endocrine hormone, we also investigated the effect of celastrol on FGF23 protein secretion. We overexpressed Myc-His-tagged rat FGF23 in UMR-106 cells as previously reported [11]. Celastrol decreased the secretion of FGF23 protein into the cell culture medium (Fig. **3C**), suggesting potential post-translational regulation.

### 3.4 Celastrol Suppresses STAT3 and FGF23 Expression *In vivo* and Alleviates CKD Kidney Injury

To determine whether celastrol inhibits STAT3 and FGF23 expression *in vivo*, we used 5/6 nephrectomized (Nx) rats as a model of CKD. As we previously reported, serum Pi levels were significantly elevated, along with increases in 24-h proteinuria, blood urea nitrogen, serum creatinine, and triglyceride levels [31]. The serum FGF23 levels remarkably increased in the 5/6 Nx group (Fig. **4A**). Celastrol exhibited renal-protective effects on 5/6 Nx rats [31]. The serum concentration of FGF23 was significantly reduced after celastrol treatment (Fig. **4A**), along with a partial recovery of renal function in the CKD rats. At present, the main source of serum FGF23 is generally considered to be the bone. Thus, we extracted RNA from rat femurs to detect the expression of STAT3 and FGF23. RT-qPCR results showed that celastrol reduced STAT3 and FGF23 expression in CKD rat femurs (Figs. **4B** and **C**). This finding suggested that celastrol might also decrease STAT3 and FGF23 expression *in vivo*.

## 4 DISCUSSION

In the end-stage of CKD, elevated FGF23 levels independently increase the risk of death and have been proposed as an early biomarker of disordered phosphorus metabolism [32, 33]. Elevated circulating levels of FGF23 contribute to the induction of cardiac hypertrophy and fibrosis, the upregulation of inflammatory cytokines in the liver, and the failure of neutrophil recruitment [34-36]. However, there are currently no effective methods to reduce serum FGF23 levels. Clinically, lowering serum Pi levels reduces circulating FGF23 levels [28, 37], suggesting a potential mutual regulatory relationship between Pi and FGF23. Since FGF23 is a hormone primarily secreted by osteoblasts [38], we utilized the rat osteosarcoma cell line UMR-106 as a model. UMR-106 cells express key osteoblastic markers (including transcription factor Runx2, extracellular matrix protein osteopontin, and functional receptor Pth1r) and respond to canonical regulators (including PTH and 1,25-dihydroxyvitamin D) [39-43]. Furthermore, it is a well-established, extensively used *in vitro* model for research on FGF23 expression [11, 44-46]. Thus, UMR-106 is a well-suited model for our research.

Inorganic Pi is essential for numerous biological processes, including energy transduction, cellular structure, and the replication of genetic information [47-49]. To maintain Pi balance, organisms must detect both environmental and internal phosphate levels. In mammals, extracellular phosphate is now recognized to directly regulate diverse cellular functions across various tissues [50-52]. Our data showed that FGF23 expression was altered in response to changes in extracellular Pi levels. In contrast, an equimolar concentration of sodium sulfate did not affect FGF23 mRNA levels, indicating that Pi-mediated transcriptional regulation is not a consequence of altered osmolality. Hori *et al*. [29] reported that Pi directly enhanced FGF23 transcription by stimulating NADPH-induced reactive oxygen species production and the MEK-ERK pathway in osteoblasts. Takashi *et al*. [11] revealed that extracellular Pi reduced proteolytic degradation of FGF23 by activating unliganded FGF receptor. They found no evidence for Pi-mediated FGF23 transcriptional regulation in UMR-106 cells, whereas our data showed that FGF23 expression was changed in response to alterations in extracellular Pi levels, possibly due to variations in cell handling or culture protocols.

The JAK/STAT signaling pathway is a crucial intracellular cascade that mediates cytokine-driven transduction of extracellular signals into the nucleus, thereby regulating gene expression involved in cell proliferation, differentiation, apoptosis, and immune responses [53-55]. Fuente *et al*. [56] reported that Jak1^S645P+/-^ mice exhibited altered mineral metabolism due to constitutive JAK1/STAT3 activation, including elevated FGF23 levels. In the liver, activation of the JAK1/STAT3 pathway promotes inflammation and upregulates FGF23 synthesis [57]. In osteoblasts, oncostatin M, a member of the interleukin-6 (IL-6) family, dose-dependently up-regulates FGF23 expression and protein secretion through gp130, as well as STAT3 and MEK1/2 [58]. There are multiple STAT3-binding sites in the FGF23 promoter [59]. Studies have reported that STAT3, a downstream molecule of IL-6 signaling, promoted FGF23 expression in calvaria organ cultures and UMR-106 cells. IL-6 enhanced FGF23 promoter activity through STAT3 phosphorylation and its evolutionarily conserved element within the FGF23 promoter [27]. Accumulating evidence suggests that FGF23 also regulates STAT3. It has been reported that FGF23 contributed to osteoporosis progression *via* the JAK-STAT pathway [60]. FGF23 can activate both the STAT3 and SMAD3 signaling pathways by increasing intracellular reactive oxygen species production in atrial fibrillation [61]. In this study, STAT3 activation was observed after Pi stimulation in the UMR-106 osteoblasts. Furthermore, pharmacological inhibition of STAT3 signaling attenuated the transcriptional activity of the FGF23 gene promoter, resulting in a marked reduction of FGF23 mRNA levels.

Current evidence demonstrated that Pi activated the ERK (extracellular regulated protein kinases) pathway *via* FGFR1 phosphorylation, leading to increased intact FGF23 production in osteocytes [11]. FGFR1 is known to dimerize with α-Klotho, to enable the binding of FGF23 [62-64]. It has been reported that unliganded FGFR1c can be activated by high Pi, although the precise mechanism remains unknown [11]. While the ERK pathway was confirmed to be activated by high Pi *via* FGFR1c, the potential involvement of other downstream signaling pathways, such as AKT and PLCγ, cannot be ruled out [11, 65-67]. In inflammation and cancers, ERK phosphorylates STAT3 to enhance its transcriptional activity [68, 69]. The interplay between ERK and STAT3 suggests a potential cross-regulatory relationship in Pi-induced FGF23 expression, which warrants further validation.

Celastrol, a natural compound, has been demonstrated to suppress STAT3 activity [70, 71]. In previous studies, surface plasmon resonance, proteome microarrays, and competitive binding assays were used to identify the molecular target of celastrol. The data showed that celastrol directly bound to STAT3, inhibiting its phosphorylation and nuclear translocation [30]. Accumulating evidence indicates that celastrol exerts potent renoprotective activity in CKD [72-74]. We previously demonstrated that celastrol ameliorates kidney injury and inflammation in 5/6 Nx rats *via* inhibiting epithelial-mesenchymal transition and TGF-β1/Smad3 pathway [31]. Treatment with celastrol significantly reduced elevated levels of 24-h proteinuria, blood urea nitrogen, serum creatinine, triglycerides, serum Pi, renal index, and cholesterol, while concurrently increasing depressed serum albumin levels. Additionally, celastrol attenuates diabetic nephropathy by upregulating SIRT1-mediated inhibition of EZH2-related wnt/β-catenin signaling [75]. In this study, we found that celastrol treatment decreased serum FGF23 levels as well as the expression of FGF23 and STAT3 genes in the femur of rats. At the same time, we also verified that celastrol suppressed transcriptional activity of the FGF23 promoter in Pi-treated osteoblasts by inhibiting STAT3 phosphorylation and nuclear translocation. As the secreted FGF23 in cell culture medium was undetectable, we overexpressed Myc-His- tagged FGF23 to investigate the inhibitory effect of celastrol on FGF23 expression. Interestingly, celastrol decreased FGF23 secretion in UMR-106 cells. These findings implied that celastrol could modulate FGF23 at the translational or post-translational level, which will be explored in future work.

Additionally, several limitations should be considered in our study. First, regarding the experimental design, the celastrol concentration was not optimized for *in vivo* conditions, and our RT-qPCR analysis relied on a single reference gene. Furthermore, utilizing deficient or knockout animal models would provide more compelling evidence to elucidate the mechanism by which celastrol inhibits STAT3 activation. Finally, although our findings indicated that high Pi activated STAT3, the precise mechanism remains unclear and will be further investigated in subsequent research.

## CONCLUSION

In summary, we found that high Pi activated STAT3 and induced FGF23 expression in osteoblasts. Meanwhile, celastrol can inhibit STAT3 activation and nuclear translocation and reduce FGF23 expression. Furthermore, in 5/6 Nx rats, celastrol intervention downregulated skeletal STAT3 expression, decreased serum FGF23 levels, and alleviated kidney injury. Therefore, celastrol could be a therapeutic agent used to decrease serum FGF23 by targeting STAT3 in CKD. We anticipate that the findings of this study will offer valuable insights into the mechanism underlying FGF23 expression in response to extracellular Pi and provide an effective means to regulate FGF23 production for the prevention and treatment of CKD. The specific molecular mechanism by which Pi activates STAT3, along with further pharmacodynamic validation of celastrol *in vivo*, will be investigated in future studies.

## Figures and Tables

**Fig. (1):**
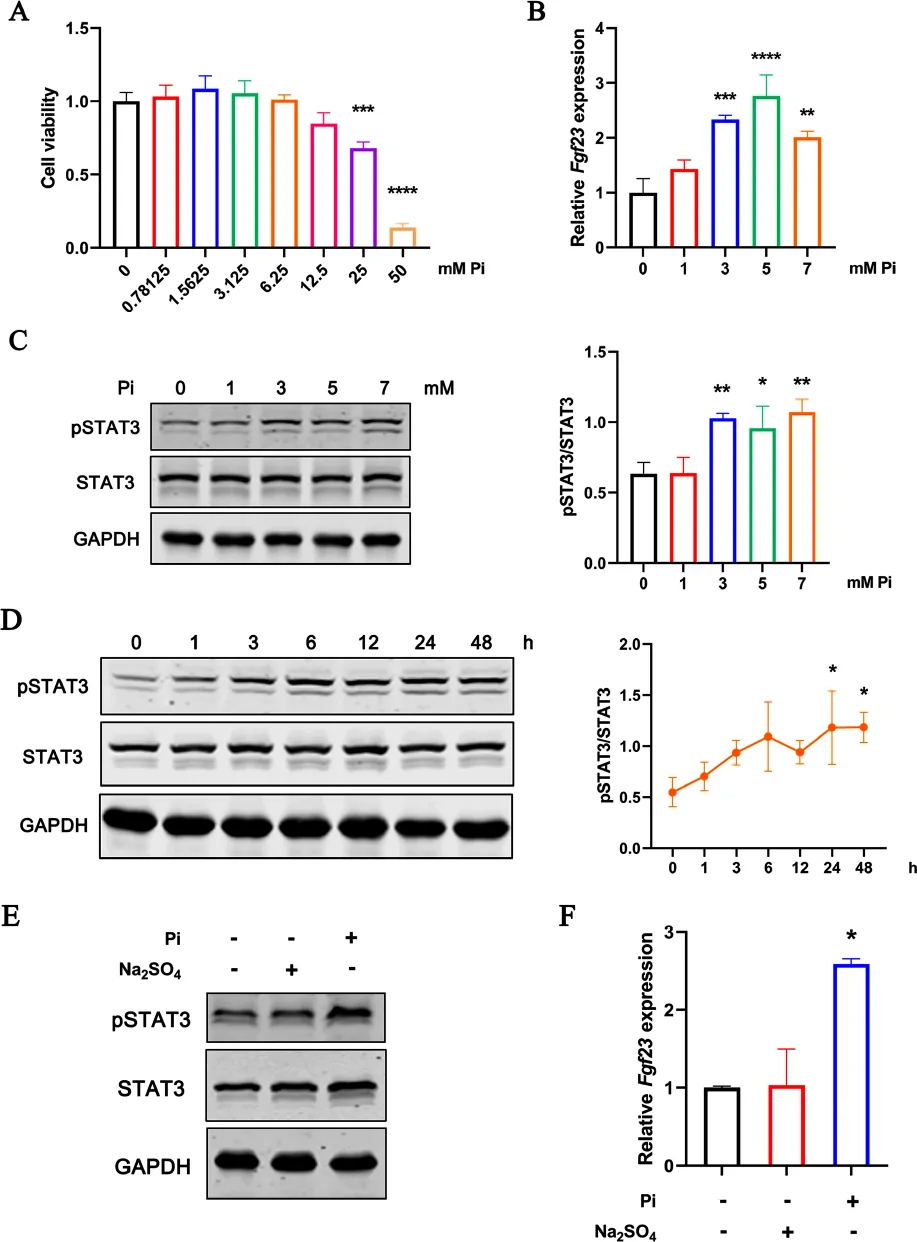
Extracellular phosphate (Pi) increased the expression of FGF23 and phosphorylated STAT3 (pSTAT3) in UMR-106 cells. (**A**) CCK-8 assay examined the cytotoxicity of different concentrations of Pi for 24 hours (n=3). (**B**) After 24 hours of stimulation with different concentrations of Pi, the FGF23 mRNA levels of UMR-106 were detected by RT-qPCR (n=3). (**C**) The protein expression of pSTAT3 after treating with gradient doses of Pi for 24 h and corresponding grayscale analysis (n=3). (**D**) The protein expression of pSTAT3 after treating with 3 mM Pi at different times and corresponding grayscale analysis (n=3). (**E**) The protein expression of pSTAT3 after treating with 3 mM Pi or 3 mM Na_2_SO_4_ for 24 h. (**F**) After 24 h stimulation of 3 mM Pi or 3 mM Na_2_SO_4_, the FGF23 mRNA levels of UMR-106 were detected by RT-qPCR (n=3). Data are presented as mean ± standard deviation of three independent experiments. *p < 0.05, **p < 0.01, ***p < 0.001, ****p < 0.0001 compared to the 0 mM Pi group.

**Fig. (2):**
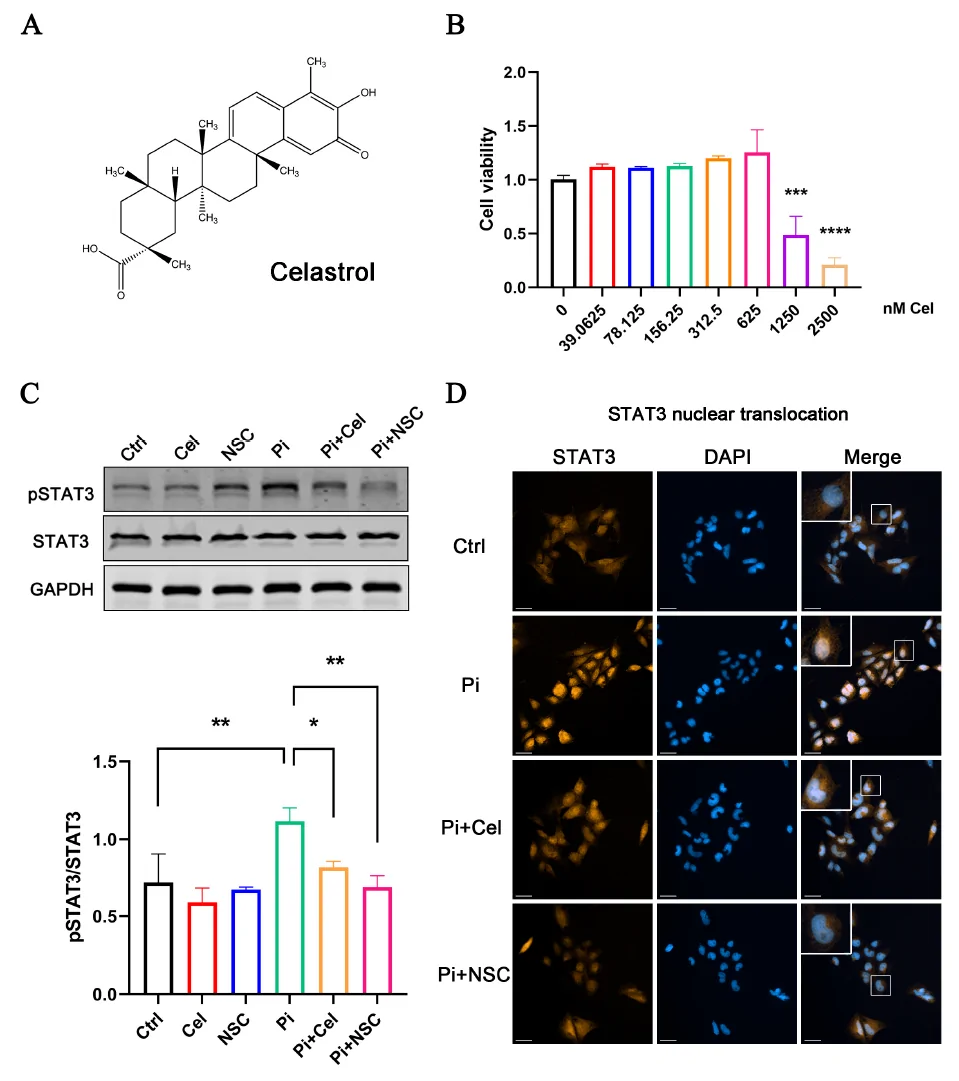
Celastrol attenuated the activation and nuclear translocation of STAT3 in UMR-106 cells. (**A**) Chemical structure of celastrol. (**B**) CCK-8 assay examined the cytotoxicity of different concentrations of celastrol for 24 h (n=3). (**C**) The protein expression of pSTAT3 after different treatments for 24 h and corresponding grayscale analysis (n=3; Ctrl: control; Pi: phosphate, 3 mM; Cel: celastrol, 300 nM; NSC: STAT3 inhibitor, NSC 74859, 50 μM). (**D**) Immunofluorescence staining of STAT3 after different treatments. Data are presented as mean ± standard deviation of three independent experiments. *p < 0.05, **p < 0.01, ***p < 0.001, ****p < 0.0001.

**Fig. (3):**
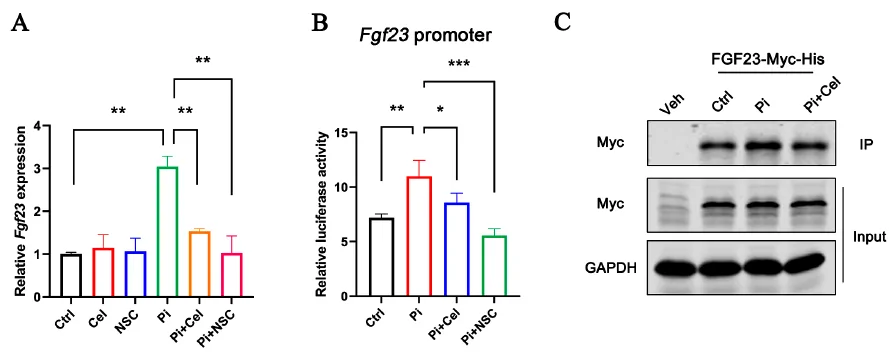
Celastrol inhibited the expression of FGF23 in UMR-106 cells. (**A**) RT-qPCR analysis of FGF23 mRNA levels after different treatments for 24 hours (n=3). (**B**) Transcriptional activity of the FGF23 promoter was detected by luciferase assays (n=3). (**C**) Immunoblotting for FGF23 secreted into the medium from UMR-106 cells overexpressing Myc-His-tagged FGF23 after immunoprecipitation with His-tag purification resin (Veh: vehicle). Data are presented as mean ± standard deviation of three independent experiments. *p < 0.05, **p < 0.01, ***p < 0.001.

**Fig. (4):**
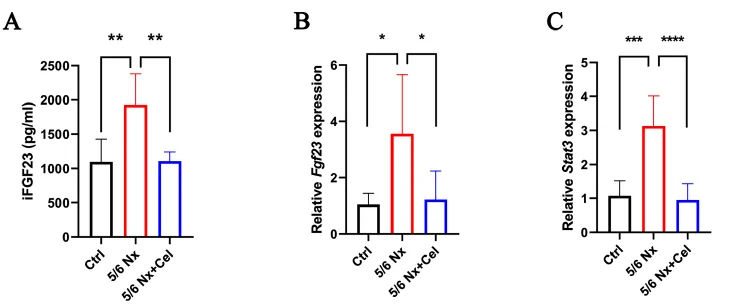
Celastrol inhibited the expression of FGF23 and STAT3 in 5/6 nephrectomized rats. (**A**) Serum FGF23 levels of each group were measured by ELISA (iFGF23: intact FGF23; Ctrl: control; 5/6 Nx: 5/6 nephrectomized; Cel: celastrol). (**B**) RT-qPCR analysis of FGF23 mRNA levels in femurs of each group. (**C**) RT-qPCR analysis of STAT3 mRNA levels in femurs of each group. Data are presented as mean ± standard deviation of six independent animals. *p < 0.05, **p < 0.01, ***p < 0.001, ****p < 0.0001.

**Table 1:** Sequence of primers for RT-qPCR.

**Primer**	**Forward Sequence (5’ to 3’)**	**Reverse Sequence (5’ to 3’)**
FGF23	ATCAGAGGATGCTGGCTCCGTAG	TAGCCGTTCTCTAGCGTCCACTG
STAT3	CTGACAGATGTTGGAGATCACC	TGTGATGCCTCCTTGATTGTC
*Gapdh*	ACCACAGTCCATGCCATCAC	TCCACCACCCTGTTGCTGTA

## Data Availability

The data sets used and/or analysed during this study are available from the corresponding author upon request.

## LIST OF ABBREVIATIONS

Pi: Phosphate
FGF23: Fibroblast Growth Factor 23
STAT3: Signal Transducer and Activator of Transcription 3
ERK: Extracellular Regulated Protein Kinases
CKD: Chronic Kidney Disease
Cel: Celastrol
RT-qPCR: Real-Time Quantitative PCR
CCK-8: Cell Counting Kit-8
ELISA: Enzyme-Linked Immunosorbent Assay
Nx: Nephrectomized
FBS: Fetal Bovine Serum
Ctrl: Control

## References

[1] Yang C., Gao B., Wang F., Chu H., Wang J., Sun X., Zhang P., Xu H., Mao H., Xing C., Chen M., Zuo L., Wang Y., Yu F., Zhang H., Wang H., Chen J., Zhang L., Zhao M.H. (2025). Executive summary for the China Kidney Disease Network (CK-NET) 2017–2018 annual data report.. Kidney Int..

[2] Isakova T., Xie H., Yang W., Xie D., Anderson A.H., Scialla J., Wahl P., Gutiérrez O.M., Steigerwalt S., He J., Schwartz S., Lo J., Ojo A., Sondheimer J., Hsu C.Y., Lash J., Leonard M., Kusek J.W., Feldman H.I., Wolf M. (2011). Fibroblast growth factor 23 and risks of mortality and end-stage renal disease in patients with chronic kidney disease.. JAMA.

[3] Donate-Correa J., Martín-Núñez E., Hernández-Carballo C., González-Luis A., Mora-Fernández C., Martín-Olivera A., Rodríguez-Ramos S., Cerro-López P., López-Castillo Á., Delgado- Molinos A., López-Tarruella V.C., Navarro-González J.F. (2024). FGF23 as a potential pathophysiological factor in peripheral arterial disease associated with chronic kidney disease.. Int. J. Mol. Sci..

[4] Caldiroli L., Molinari P., D’Alessandro C., Cupisti A., Alfieri C., Castellano G., Vettoretti S. (2025). Osteosarcopenia in chronic kidney disease: An overlooked syndrome?. J. Cachexia Sarcopenia Muscle.

[5] Edmonston D., Grabner A., Wolf M. (2024). FGF23 and klotho at the intersection of kidney and cardiovascular disease.. Nat. Rev. Cardiol..

[6] Sun T., Yu X. (2023). FGF23 actions in CKD-MBD and other organs during CKD.. Curr. Med. Chem..

[7] Agoro R., White K.E. (2023). Regulation of FGF23 production and phosphate metabolism by bone–kidney interactions.. Nat. Rev. Nephrol..

[8] Raju S., Saxena R. (2025). Hyperphosphatemia in kidney failure: Pathophysiology, challenges, and critical role of phosphorus management.. Nutrients.

[9] Saito H., Maeda A., Ohtomo S., Hirata M., Kusano K., Kato S., Ogata E., Segawa H., Miyamoto K., Fukushima N. (2005). Circulating FGF-23 is regulated by 1alpha,25-dihydroxyvitamin D3 and phosphorus *in vivo*.. J. Biol. Chem..

[10] Gerber J.S., Arroyo E.M.P., Pastor J., Correia M., Rudloff S., Moe O.W., Egli-Spichtig D., Mohebbi N., Wagner C.A. (2025). Controlled dietary phosphate loading in healthy young men elevates plasma phosphate and FGF23 levels.. Pflugers Arch..

[11] Takashi Y., Kosako H., Sawatsubashi S., Kinoshita Y., Ito N., Tsoumpra M.K., Nangaku M., Abe M., Matsuhisa M., Kato S., Matsumoto T., Fukumoto S. (2019). Activation of unliganded FGF receptor by extracellular phosphate potentiates proteolytic protection of FGF23 by its O-glycosylation.. Proc. Natl. Acad. Sci. USA.

[12] Khoshniat S., Bourgine A., Julien M., Weiss P., Guicheux J., Beck L. (2011). The emergence of phosphate as a specific signaling molecule in bone and other cell types in mammals.. Cell. Mol. Life Sci..

[13] Lee S.H., Kim K.H., Lee S.M., Park S.J., Lee S., Cha R.H., Lee J.W., Kim D.K., Kim Y.S., Ye S.K., Yang S.H. (2024). STAT3 blockade ameliorates LPS-induced kidney injury through macrophage-driven inflammation.. Cell Commun. Signal..

[14] Han X., Zhang C., Lei Q., Xu J., Zhou Y. (2025). Stiffness regulates extracellular matrix synthesis in fibroblasts by DDR1-TGF-β/STAT3 mechanotransduction axis.. Biomater. Adv..

[15] Wang Y., Feng Y., Li M., Yang M., Shi G., Xuan Z., Yin D., Xu F. (2022). Traditional Chinese Medicine in the treatment of chronic kidney diseases: Theories, applications, and mechanisms.. Front. Pharmacol..

[16] Shao G., Zhu S., Yang B. (2021). Applications of herbal medicine to treat autosomal dominant polycystic kidney disease.. Front. Pharmacol..

[17] Lei H., Ruan Y., Ding R., Li H., Zhang X., Ji X., Wang Q., Lv S. (2025). The role of celastrol in inflammation and diseases.. Inflamm. Res..

[18] Yang R., Yao J., Ma H., Shui C., Li T., Zhang S., Li C. (2025). Celastrol promotes apoptotic cell death in thyroid cancer cells through a caspases-dependent pathway.. Thyroid Res..

[19] Li Y., Zhu Y., Shang F.F., Xu L., Jiang D., Sun B., Zhang L., Luo C., Zhang A., Zhang H., Ding C. (2024). Discovery of urea derivatives of celastrol as selective peroxiredoxin 1 inhibitors against colorectal cancer cells.. J. Med. Chem..

[20] Liu H., Xiang X., Shi C., Guo J., Ran T., Lin J., Dong F., Yang J., Miao H. (2025). Oxidative stress and inflammation in renal fibrosis: Novel molecular mechanisms and therapeutic targets.. Chem. Biol. Interact..

[21] Wu Q., Wang J., Wang Y., Xiang L., Tan Y., Feng J., Zhang Z., Zhang L. (2022). Targeted delivery of celastrol to glomerular endothelium and podocytes for chronic kidney disease treatment.. Nano Res..

[22] Yang X., Chen A., Liang Q., Dong Q., Fu M., Liu X., Wang S., Li Y., Ye Y., Lan Z., Ou J.S., Lu L., Yan J. (2021). Up-regulation of heme oxygenase-1 by celastrol alleviates oxidative stress and vascular calcification in chronic kidney disease.. Free Radic. Biol. Med..

[23] Wu K., Qiu C., Ma Q., Chen F., Lu T. (2024). The anti-cancer mechanism of Celastrol by targeting JAK2/STAT3 signaling pathway in gastric and ovarian cancer.. Toxicol. Appl. Pharmacol..

[24] Percie du Sert N., Hurst V., Ahluwalia A., Alam S., Avey M.T., Baker M., Browne W.J., Clark A., Cuthill I.C., Dirnagl U., Emerson M., Garner P., Holgate S.T., Howells D.W., Karp N.A., Lazic S.E., Lidster K., MacCallum C.J., Macleod M., Pearl E.J., Petersen O.H., Rawle F., Reynolds P., Rooney K., Sena E.S., Silberberg S.D., Steckler T., Würbel H. (2020). The ARRIVE guidelines 2.0: Updated guidelines for reporting animal research.. PLoS Biol..

[25] Kannaiyan R., Shanmugam M.K., Sethi G. (2011). Molecular targets of celastrol derived from Thunder of God Vine: Potential role in the treatment of inflammatory disorders and cancer.. Cancer Lett..

[26] Zhou Q., Guo X., Chen T., Liu Y., Ji H., Sun Y., Yang X., Ouyang C., Liu X., Lei M. (2024). The neuroprotective role of celastrol on hippocampus in diabetic rats by inflammation restraint, insulin signaling adjustment, Aβ reduction and synaptic plasticity alternation.. Biomed. Pharmacother..

[27] Durlacher-Betzer K., Hassan A., Levi R., Axelrod J., Silver J., Naveh-Many T. (2018). Interleukin-6 contributes to the increase in fibroblast growth factor 23 expression in acute and chronic kidney disease.. Kidney Int..

[28] Tsai W.C., Wu H.Y., Peng Y.S., Hsu S.P., Chiu Y.L., Chen H.Y., Yang J.Y., Ko M.J., Pai M.F., Tu Y.K., Hung K.Y., Chien K.L. (2018). Effects of lower *versus* higher phosphate diets on fibroblast growth factor-23 levels in patients with chronic kidney disease: A systematic review and meta-analysis.. Nephrol. Dial. Transplant..

[29] Hori M., Kinoshita Y., Taguchi M., Fukumoto S. (2016). Phosphate enhances FGF23 expression through reactive oxygen species in UMR-106 cells.. J. Bone Miner. Metab..

[30] Ye S., Luo W., Khan Z.A., Wu G., Xuan L., Shan P., Lin K., Chen T., Wang J., Hu X., Wang S., Huang W., Liang G. (2020). Celastrol attenuates angiotensin II–induced cardiac remodeling by targeting STAT3.. Circ. Res..

[31] Tang Y.W., Yang R.C., Wan F., Tang X.L., Zhang H.Q., Lin Y. (2022). Celastrol attenuates renal injury in 5/6 nephrectomized rats *via* inhibiting epithelial–mesenchymal transition and transforming growth factor-β1/Smad3 pathway.. Exp. Biol. Med..

[32] Isakova T., Wahl P., Vargas G.S., Gutiérrez O.M., Scialla J., Xie H., Appleby D., Nessel L., Bellovich K., Chen J., Hamm L., Gadegbeku C., Horwitz E., Townsend R.R., Anderson C.A.M., Lash J.P., Hsu C., Leonard M.B., Wolf M. (2011). Fibroblast growth factor 23 is elevated before parathyroid hormone and phosphate in chronic kidney disease.. Kidney Int..

[33] Jansson K.P., Yu A.S.L., Stubbs J.R. (2022). Contribution of phosphate and FGF23 to CKD progression.. Curr. Opin. Nephrol. Hypertens..

[34] Richter B., Faul C. (2018). FGF23 actions on target tissues - With and without klotho.. Front. Endocrinol..

[35] Deng C., Wu Y. (2025). Vitamin D–parathyroid hormone–fibroblast growth factor 23 axis and cardiac remodeling.. Am. J. Cardiovasc. Drugs.

[36] Mattinzoli D., Li M., Castellano G., Ikehata M., Armelloni S., Elli F.M., Molinari P., Tsugawa K., Alfieri C.M., Messa P. (2022). Fibroblast growth factor 23 level modulates the hepatocyte’s alpha-2-HS-glycoprotein transcription through the inflammatory pathway TNFα/NFκB.. Front. Med..

[37] Rodelo-Haad C., Rodríguez-Ortiz M.E., Garcia-Sáez R., Rivas- Domínguez A., Jurado-Montoya D., Martín-Malo A., Rodríguez M., Pendón-Ruiz de Mier M.V., Muñoz-Castañeda J.R. (2025). The true cost of phosphate control in chronic kidney disease.. Clin. Kidney J..

[38] Lai S., Perrotta A.M., Tartaglione L., Mastroluca D., Tinti F., Menè P., Pasquali M., Ferraro P.M., Mazzaferro S., Rotondi S. (2025). Chronic kidney disease - Mineral and bone disorder in autosomal dominant policystic kidney disease.. Bone.

[39] Teerapornpuntakit J., Wongdee K., Krishnamra N., Charoenphandhu N. (2016). Expression of osteoclastogenic factor transcripts in osteoblast-like UMR-106 cells after exposure to FGF-23 or FGF-23 combined with parathyroid hormone.. Cell Biol. Int..

[40] Wejheden C., Brunnberg S., Hanberg A., Lind P.M. (2006). Osteopontin: A rapid and sensitive response to dioxin exposure in the osteoblastic cell line UMR-106.. Biochem. Biophys. Res. Commun..

[41] Kim B.H., Wang F.I., Pereverzev A., Chidiac P., Dixon S.J. (2019). Toward defining the pharmacophore for positive allosteric modulation of PTH1 receptor signaling by extracellular nucleotides.. ACS Pharmacol. Transl. Sci..

[42] Martorano A.S., Neto W.R., Teixeira L.N., Bordignon C.M., de Oliveira P.T., de Castro Raucci L.M.S. (2025). Impact of Ilex paraguariensis extract on osteoblastic phenotype expression.. Mol. Biol. Rep..

[43] Guerrero R., de la Piedra C., Teixeiro E., Bragado R., Traba M.L. (2004). Hydrocortisone and deflazacort induce different effects on vitamin D receptor level increase produced by 1,25-dihydroxyvitamin D3 in rat osteoblast-like UMR-106 cells.. J. Rheumatol..

[44] Radvanyi Z., Yoo E.J., Kandasamy P., Salas-Bastos A., Monnerat S., Refardt J., Christ-Crain M., Hayashi H., Kondo Y., Jantsch J., Rubio-Aliaga I., Sommer L., Wagner C.A., Hediger M.A., Kwon H.M., Loffing J., Pathare G. (2024). Extracellular sodium regulates fibroblast growth factor 23 (FGF23) formation.. J. Biol. Chem..

[45] Tamamura Y., Terai K., Yamaguchi A. (2025). Notch signaling modulates FGF23 expression through crosstalk with hypoxia and PTH pathways in osteogenic cells.. Mol. Cell. Endocrinol..

[46] Hum J.M., O’Bryan L.M., Tatiparthi A.K., Cass T.A., Clinkenbeard E.L., Cramer M.S., Bhaskaran M., Johnson R.L., Wilson J.M., Smith R.C., White K.E. (2017). Chronic hyperphosphatemia and vascular calcification are reduced by stable delivery of soluble klotho.. J. Am. Soc. Nephrol..

[47] Dietrich L., Agip A.N.A., Kunz C., Schwarz A., Kühlbrandt W. (2024). *In situ* structure and rotary states of mitochondrial ATP synthase in whole *Polytomella* cells.. Science.

[48] Davidson J.W., Jain R., Kizzar T., Geoghegan G., Nesbitt D.J., Cavanagh A., Abe A., Nyame K., Hunger A., Chao X., James I., Walesewicz H., Baldwin D.A., Wade G., Michorowska S., Verma R., Schueler K., Hinkovska-Galcheva V., Shishkova E., Ding W.X., Coon J.J., Shayman J.A., Abu-Remaileh M., Simcox J.A. (2025). Hepatic lipid remodeling in cold exposure uncovers direct regulation of bis(monoacylglycero)phosphate lipids by phospholipase A2 group XV.. Cell Metab..

[49] Hernando N., Gagnon K., Lederer E. (2021). Phosphate transport in epithelial and nonepithelial tissue.. Physiol. Rev..

[50] Chande S., Bergwitz C. (2018). Role of phosphate sensing in bone and mineral metabolism.. Nat. Rev. Endocrinol..

[51] Centeno P.P., Herberger A., Mun H.C., Tu C., Nemeth E.F., Chang W., Conigrave A.D., Ward D.T. (2019). Phosphate acts directly on the calcium-sensing receptor to stimulate parathyroid hormone secretion.. Nat. Commun..

[52] Michigami T., Yamazaki M., Razzaque M.S. (2022). Extracellular phosphate, inflammation and cytotoxicity.. Adv. Exp. Med. Biol..

[53] Lv Y., Qi J., Babon J.J., Cao L., Fan G., Lang J., Zhang J., Mi P., Kobe B., Wang F. (2024). The JAK-STAT pathway: From structural biology to cytokine engineering.. Signal Transduct. Target. Ther..

[54] Maslah N., Kaci N., Roux B., Alexe G., Marie R., Pasquer H., Verger E., De Oliveira R.D., Culeux C., Mlayah B., Gauthier N., Gonzales F., Zhao L.P., Ganesan S., Gou P., Ling F., Soret-Dulphy J., Parquet N., Vainchenker W., Raffoux E., Padua R.A., Giraudier S., Marty C., Plo I., Lobry C., Stegmaier K., Puissant A., Kiladjian J.J., Cassinat B., Benajiba L. (2025). JAK2 inhibition mediates clonal selection of RAS pathway mutations in myeloproliferative neoplasms.. Nat. Commun..

[55] Yang Z., Hu Y., Yang C., Li Y., Zhong X., Wu Z., Zuo Y., Gan S., Chen L., Zeng Z., Qi G., Chen Y., Zhou J., Zhang X.J., Li H., Yi B. (2025). SMYD2-mediated methylation of STAT1 protects against hepatic ischaemia/reperfusion injury by blocking JAK-STAT1 signalling pathways.. Gut.

[56] Fuente R., Gehring N., Bettoni C., Gil-Peña H., Alonso-Durán L., Michalke B., Santos F., Wagner C.A., Rubio-Aliaga I. (2021). Systemic Jak1 activation causes extrarenal calcitriol production and skeletal alterations provoking stunted growth.. FASEB J..

[57] Daryadel A., Ruiz P.A., Gehring N., Stojanovic D., Ugrica M., Bettoni C., Sabrautzki S., Pastor-Arroyo E.M., Frey-Wagner I., Lorenz-Depiereux B., Strom T.M., de Angelis M.H., Rogler G., Wagner C.A., Rubio-Aliaga I. (2021). Systemic Jak1 activation provokes hepatic inflammation and imbalanced FGF23 production and cleavage.. FASEB J..

[58] Münz S., Feger M., Föller M. (2023). Oncostatin M is a regulator of fibroblast growth factor 23 (FGF23) in UMR106 osteoblast-like cells.. Sci. Rep..

[59] Saini R.K., Kaneko I., Jurutka P.W., Forster R., Hsieh A., Hsieh J.C., Haussler M.R., Whitfield G.K. (2013). 1,25-dihydroxyvitamin D(3) regulation of fibroblast growth factor-23 expression in bone cells: Evidence for primary and secondary mechanisms modulated by leptin and interleukin-6.. Calcif. Tissue Int..

[60] Xu L., Zhang L., Zhang H., Yang Z., Qi L., Wang Y., Ren S. (2018). The participation of fibroblast growth factor 23 (FGF23) in the progression of osteoporosis *via* JAK/STAT pathway.. J. Cell. Biochem..

[61] Dong Q., Li S., Wang W., Han L., Xia Z., Wu Y., Tang Y., Li J., Cheng X. (2019). FGF23 regulates atrial fibrosis in atrial fibrillation by mediating the STAT3 and SMAD3 pathways.. J. Cell. Physiol..

[62] Ji L., Kong R., Yu Y., Wan W., Zhao D., Gao J. (2025). Klotho promotes bone formation in rheumatoid arthritis-associated osteoporosis by modulating the FGF23/Fgfr1/NF-κB pathway and inhibiting ferroptosis.. Mol. Immunol..

[63] Yanucil C., Kentrup D., Campos I., Czaya B., Heitman K., Westbrook D., Osis G., Grabner A., Wende A.R., Vallejo J., Wacker M.J., Navarro-Garcia J.A., Ruiz-Hurtado G., Zhang F., Song Y., Linhardt R.J., White K., Kapiloff M.S., Faul C. (2022). Soluble α-klotho and heparin modulate the pathologic cardiac actions of fibroblast growth factor 23 in chronic kidney disease.. Kidney Int..

[64] Chen L., Fu L., Sun J., Huang Z., Fang M., Zinkle A., Liu X., Lu J., Pan Z., Wang Y., Liang G., Li X., Chen G., Mohammadi M. (2023). Structural basis for FGF hormone signalling.. Nature.

[65] Yamazaki M., Ozono K., Okada T., Tachikawa K., Kondou H., Ohata Y., Michigami T. (2010). Both FGF23 and extracellular phosphate activate Raf/MEK/ERK pathway *via* FGF receptors in HEK293 cells.. J. Cell. Biochem..

[66] Bollenbecker S., Hirsch M.J., Matthews E.L., Easter M., Vang S., Howze P.H., Morales A.N., Harris E., Barnes J.W., Faul C., Krick S. (2024). Chronic kidney disease–associated lung injury is mediated by phosphate-induced MAPK/AKT signaling.. Am. J. Respir. Cell Mol. Biol..

[67] Kawai M., Kinoshita S., Ozono K., Michigami T. (2016). Inorganic phosphate activates the AKT/mTORC1 pathway and shortens the life span of an α-klotho-deficient model.. J. Am. Soc. Nephrol..

[68] Decker T., Kovarik P. (2000). Serine phosphorylation of STATs.. Oncogene.

[69] Wang Z., Zhao P., Tian K., Qiao Z., Dong H., Li J., Guan Z., Su H., Song Y., Ma X. (2024). TMEM9 promotes lung adenocarcinoma progression *via* activating the MEK/ERK/STAT3 pathway to induce VEGF expression.. Cell Death Dis..

[70] Xiang S., Chen J., Deng M., Wang Z., Li X., Lin D., Zhou J. (2024). Celastrol ameliorates experimental autoimmune uveitis through STAT3 targeting and gut microenvironment reprofiling.. Int. Immunopharmacol..

[71] Li G., Zhou L., Deng H., Huang C., Wang N., Yue L., Zhang P., Zhou Y., Zhou W., Gao Y. (2022). Targeting OPA1-mediated mitochondrial fusion contributed to celastrol’s anti-tumor angiogenesis effect.. Pharmaceutics.

[72] Rahman M.A., Akter S., Dorotea D., Mazumder A., Uddin M.N., Hannan M.A., Hossen M.J., Ahmed M.S., Kim W., Kim B., Uddin M.J. (2022). Renoprotective potentials of small molecule natural products targeting mitochondrial dysfunction.. Front. Pharmacol..

[73] Pan M., Wang Z., Wang Y., Jiang X., Fan Y., Gong F., Sun Y., Wang D. (2023). Celastrol alleviated acute kidney injury by inhibition of ferroptosis through Nrf2/GPX4 pathway.. Biomed. Pharmacother..

[74] Al-Tantawy S.M., Eraky S.M., Eissa L.A. (2025). Novel therapeutic target for diabetic kidney disease through downregulation of miRNA-192-5p and miRNA-21-5p by celastrol: Implication of autophagy, oxidative stress, and fibrosis.. Naunyn Schmiedebergs Arch. Pharmacol..

[75] Tang Y., Wan F., Tang X., Lin Y., Zhang H., Cao J., Yang R. (2023). Celastrol attenuates diabetic nephropathy by upregulating SIRT1- mediated inhibition of EZH2 related Wnt/β-catenin signaling.. Int. Immunopharmacol..

