# Supplementary material for: Celastrol Attenuates FGF23 Expression in Osteoblasts by Inhibiting STAT3 Activation
Source: Endocr Metab Immune Disord Drug Targets. 2026 Jul 1;26:E18715303420913. doi: 10.2174/0118715303420913251205154844 (PMC13670194; doi:10.2174/0118715303420913251205154844)
Supplement: Supplementary file 1 [file EMIDDT-26-E18715303420913_SD1.pdf]

Supplementary Material

Celastrol Attenuates *Fgf23* Expression in Osteoblasts by Inhibiting STAT3 Activation

Jiawei Cao[1], Yuewen Tang[1,2], Feng Wan[1,3], Tingjiao Xu[4] and Ruchun Yang[1,3,*]

[1]Department of Nephrology, Hangzhou TCM Hospital Affiliated to Zhejiang Chinese Medical University, Hangzhou, China; [2]Department of Nephrology, Hangzhou Dingqiao Hospital, Hangzhou, China; [3]Zhejiang Key Laboratory of Blood-Stasis-Toxin Syndrome, Zhejiang Chinese Medical University, Hangzhou, China; [4]College of Life Sciences, Zhejiang Chinese Medical University, Hangzhou, China

Supplementary Table 1. Osmolarity of the cell culture medium

**Medium**	**Osmolality (mOsm/kg)**
Control medium	344.3 ± 0.6
Control medium + 3 mM Pi	346.0 ± 1.7
Control medium + 3 mM Na_2SO_4	345.0 ± 2.0
